# Rigorous methodological approaches to address knowledge gaps in exercise, nutrition, immunity, and infection risk research

**DOI:** 10.14814/phy2.70479

**Published:** 2025-07-25

**Authors:** G. Davison, C. Chidley, A. W. Jones

**Affiliations:** ^1^ School of Natural Sciences University of Kent Canterbury UK; ^2^ School of Sport and Exercise Science University of Derby Derby UK; ^3^ School of Translational Medicine Monash University Melbourne Victoria Australia

**Keywords:** clinically relevant, exercise, immune, in vivo, infection, nutrition, URTI

## Abstract

Upper respiratory tract infections (URTI) and the associated symptoms can have significant impacts for the general population and athletes (e.g., affecting training, recovery, and performance). Various factors influence the risk of URTI, including physiological stress (i.e., exercise), psychological stress, sleep, travel, nutrition, and pathogen exposure. Traditional research in exercise immunology has relied heavily on ex vivo immune markers, which lack clinical relevance and overlook immune redundancy and robustness. As such, it is unsurprising that interventions affecting these markers do not always align with URTI risk. More recently, evidence has emphasized the importance of in vivo immune markers and clinical outcomes to assess infection risk, and the role of interventions to mitigate this. Traditionally, nutritional exercise immunology research has focused only on mechanisms affecting URTI via immune modulation. However, nutritional interventions may also act via immune‐independent mechanisms (e.g., direct antipathogenic mechanisms). For future research, we recommend prioritizing clinically relevant endpoints (validated URTI logs; pathogen screening); using in vivo markers representing the integrated immune response; large sample size; and implementing stringent study controls. Experimental infection challenge models offer controlled investigations of interventions. These approaches will enhance our ability to determine the impact of exercise and nutrition on immunity and URTI outcomes in athletes.

## 1 ATHLETE INFECTION RISK

Upper respiratory tract infections (URTI) are among the most frequent presentations to General Practitioners (Hashem & Hall, 2003). They are mainly caused by viral infection, with rhinoviruses, coronaviruses, influenza viruses, adenoviruses, parainfluenza viruses, respiratory syncytial viruses, and enteroviruses being the most common (Heikkinen & Järvinen, 2003). While usually mild, they can contribute to absence from work, reduced productivity, reduced feelings of well‐being, health and quality of life, and reduced social interaction (Bramley et al., 2002; Hashem & Hall, 2003; Hellgren et al., 2010). However, for athletes, they can impair performance or compromise the ability to train and/or recover optimally (in a broader context, the term athlete may also apply to individuals that are regularly exposed to physical and/or other stressors as part of their profession for example, military personnel, emergency services).

**FIGURE 1:**
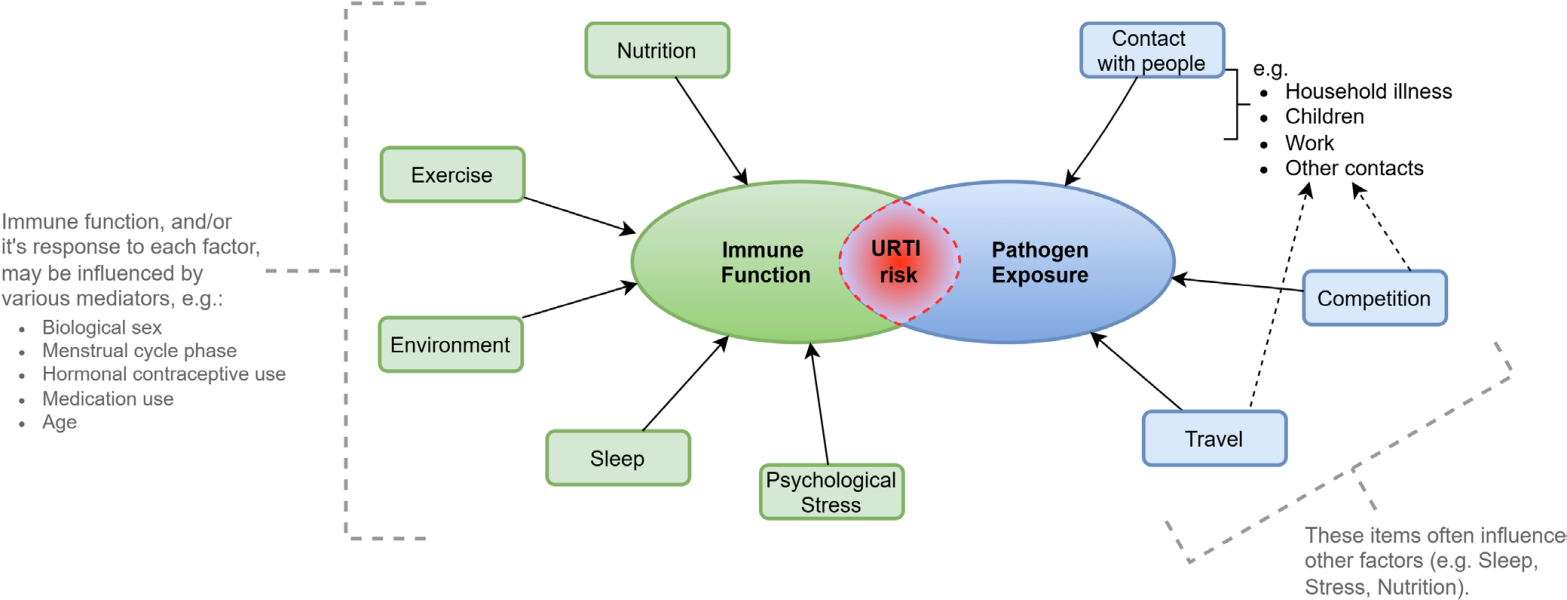
Factors that can influence URTI risk in athletes. URTI risk is influenced by the combination of host defense (immune function) and pathogen exposure; consequently, factors affecting either component can potentially alter this risk.

The average person, and many athletes, will typically experience 2–3 URTI episodes per year (Hull & Davison, 2020). Moderate exercise is generally accepted to enhance or maintain immune system functions, contributing to a reduced risk of infection (Matthews et al., 2002; Nieman et al., 1993; Nieman & Wentz, 2019). In contrast, some athletes are “illness‐prone,” experiencing URTIs more frequently. Whether classified as illness‐prone or not, such episodes may cluster around certain training periods (e.g., training camps), competition, and major events, and be considered an additional threat to an athlete's performance and/or preparation (Cannon, 1993; Engebretsen et al., 2010, 2013; Gleeson and Walsh, 2012; Raysmith & Drew, 2016; Robinson & Milne, 2002; Schwellnus et al., 2021; Valtonen et al., 2019). URTI risk is influenced by various factors, including pathogen exposure, and factors that influence host defense (immune functions). Suggested factors (summarized in Figure 1) include: the physiological stress of strenuous exercise, psychological stress (which may be training or competition related and/or general life stressors), environmental conditions, sleep, travel, and nutritional status. The relative importance and contribution of each factor has been debated (e.g., is the clustering of episodes caused by exercise‐induced immune changes, or is it caused by other factors such as increased pathogen exposure (e.g., Campbell & Turner, 2018; Simpson et al., 2020)) but it is likely an oversimplification to consider any factor in isolation, with effects being complex and multifaceted (Figure 1). Nevertheless, such debates were a timely reminder of the importance of clinically relevant outcomes, but should not detract from the fact that all of these factors likely contribute to some extent to overall URTI risk (Figure 1). It is clear that certain groups of athletes have a higher likelihood of presenting with URTI symptoms in general and/or at certain times (Engebretsen et al., 2010, 2013; Nieman & Wentz, 2019; Robinson & Milne, 2002; Svendsen et al., 2016; Valtonen et al., 2019), highlighting the importance of considering the complex interactions of all components.

## 2 RECENT ADVANCES IN EXERCISE IMMUNOLOGY RESEARCH

Traditional approaches in Exercise Immunology relied heavily on ex vivo (or in vitro) immune markers, including (e.g.) peripheral blood immune cell numbers and functions, mucosal immune markers, and systemic concentrations of regulatory factors such as cytokines and hormones. While this may provide mechanistic insights, such markers in isolation may lack clinical relevance. It is for this reason that the relative value of these markers (used in isolation) has been questioned, and such evidence (alone) is now acknowledged as lacking clinical relevance as a representation of URTI risk in the “real world” (Albers et al., 2005; Albers et al., 2013).

### 2.1 Clinically relevant outcomes and in vivo immune markers

Compared to ex vivo markers, measures of in vivo immune function are considered more “clinically relevant” outcomes of URTI risk as they assess the ability of the whole integrated system to mount a co‐ordinated response (Albers et al., 2005; Albers et al., 2013). In the exercise immunology literature, such measures include challenge‐type tests (such as Delayed Type Hypersensitivity (DTH) or Contact Hypersensitivity (CHS) tests), response to vaccination (in particular the early kinetics of the response), and possibly the reactivation of latent viruses (as determined by viral shedding and DNA concentration in saliva etc). Actual presentation with URTI episodes, is the most clinically relevant outcome, typically recorded via self‐report logs. Some questions have been raised regarding the validity of self‐reported URTI episodes (Albers et al., 2005; Albers et al., 2013; Bermon et al., 2017; Cox et al., 2008; Spence et al., 2007; Walsh et al., 2011) and few studies have attempted to determine infectious cause (Davison et al., 2025; Hanstock et al., 2016; Spence et al., 2007; Valtonen et al., 2019), with some reporting discrepancies between logs, physician, and laboratory diagnoses (Cox et al., 2008). However, it is important to note that a negative result from laboratory screening does not necessarily mean that there was no infection or the physician diagnosis was incorrect, rather the laboratory screening may have failed to detect the pathogen. Indeed, the typical procedures are targeted to specific pathogens and may be ineffective at identifying new pathogens or novel variants (Kustin et al., 2019). Indeed, Kustin et al. (Kustin et al., 2019), demonstrated that 18% (54/300) of samples from patients presenting with URTI symptoms were negative using traditional screening methods (i.e., targeted PCR pathogen panels), but a next generation sequencing (NGS) approach identified a URTI‐pathogen in all cases. This suggests that some previously reported discrepancies are explained by the screening methods employed.

## 3 IMMUNE REDUNDANCY, ROBUSTNESS AND COMPENSATION MECHANISMS: CRITICAL YET OFTEN OVERLOOKED CONCEPTS IN THE LINK BETWEEN IMMUNITY AND ILLNESS RISK

### 3.1 Immune redundancy

**FIGURE 2:**
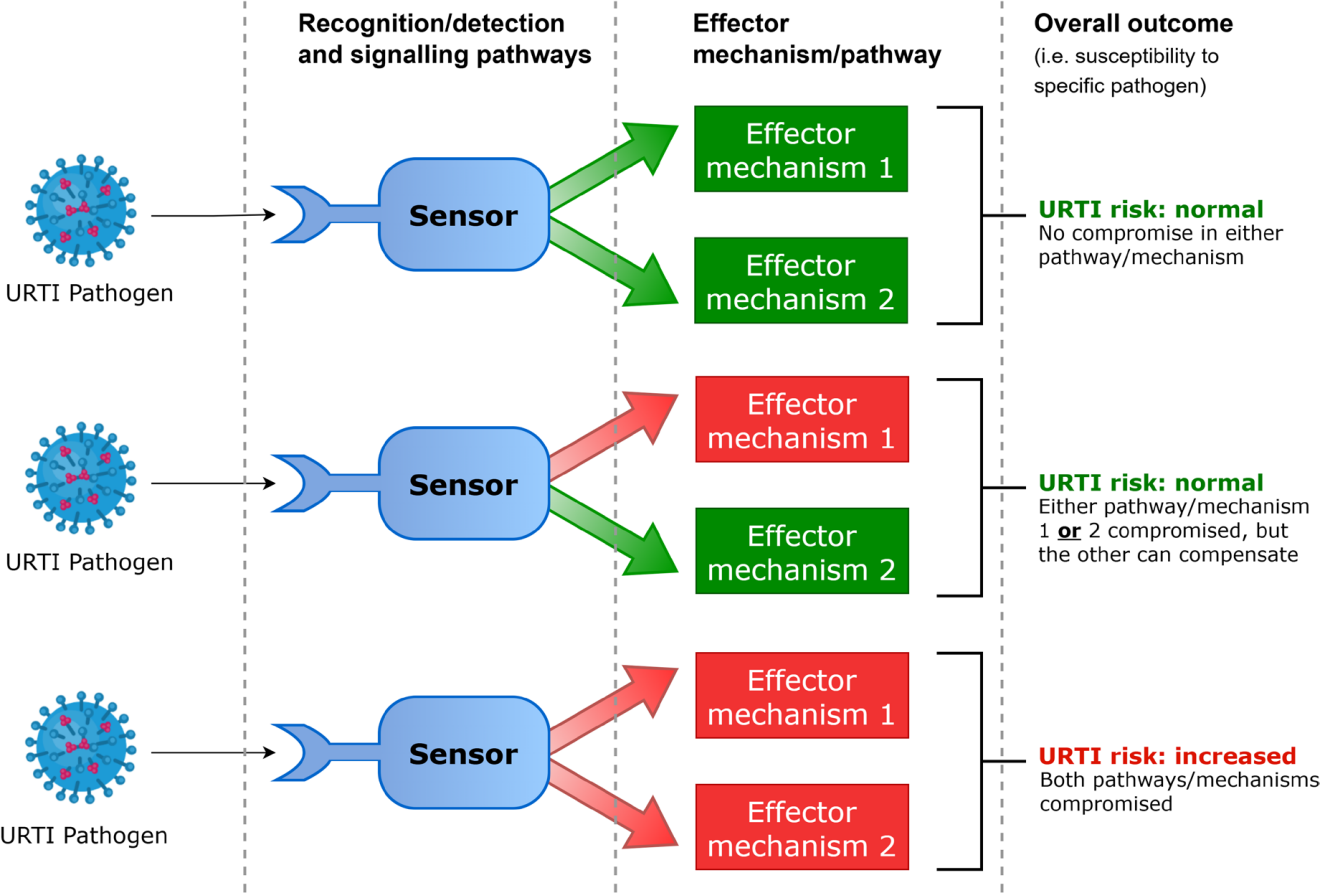
Immune redundancy and compensation concept. Green arrows and boxes indicate pathway not compromised. Red arrows and boxes indicate pathway is compromised (which may occur either at the level of the sensor, the level of the effector mechanism, or a combination). Overall outcome (infection risk) may not be adversely affected if only one pathway is compromised, and the other is able to compensate.

One reason why isolated ex vivo markers are not “clinically relevant” and do not always relate to URTI risk is that such methods overlook the complex, integrated nature of host defense, and the considerable redundancy in the immune system (i.e., overall host defense *is a product of functional interactions between different components*, Nish & Medzhitov, 2011). This means that overlapping components ensure a detriment in one component does not necessarily result in compromised “overall” defense (i.e., changes in a single marker don't often reflect true host defense status) (Figure 2). A good example of this has been reported in patients with low salivary IgA, whereby high IgM can compensate and results in no change in risk of URTI. However, individuals deficient in both IgA and IgM have a higher than “normal” URTI rate (Brandtzaeg et al., 1987).

### 3.2 Immune robustness

**FIGURE 3:**
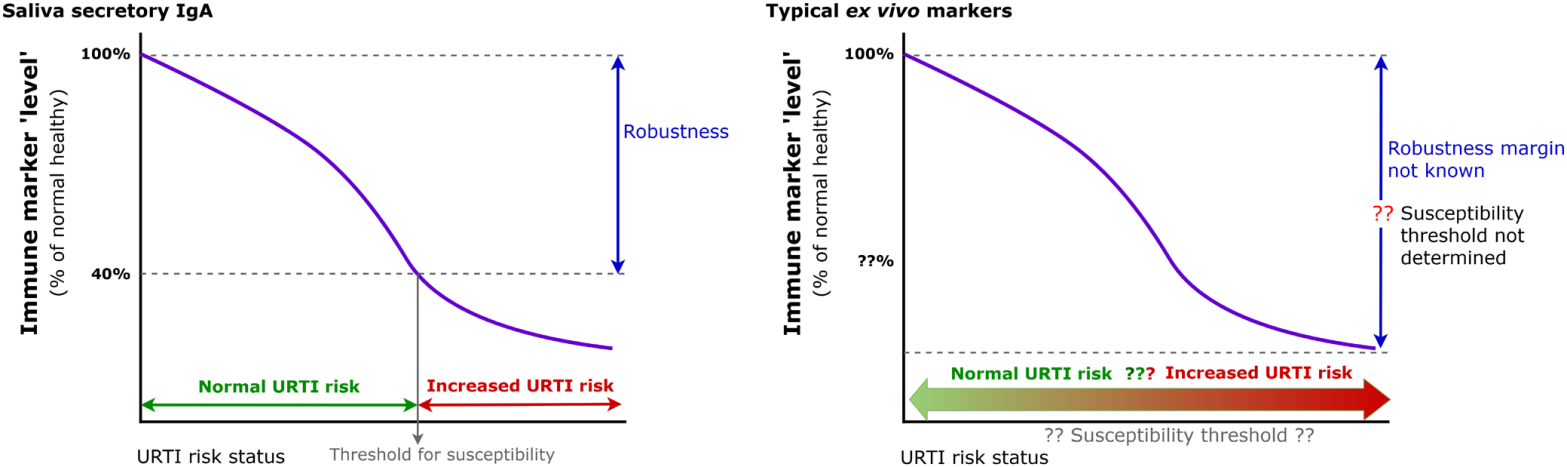
Representation of immune robustness. Left panel: example with sIgA, where a “robustness” threshold is known (point beyond which URTI risk is increased). Right panel: example with other ex vivo markers where the critical threshold, and hence size of the “robustness margin” has not been determined.

There are further issues arising when overlooking robustness of the immune system. Exercise Immunology research has tended to present changes in immune markers as meaningful, without considering the clinical significance of such changes (e.g., the magnitude of change required to influence infection risk). This question is often overlooked, but the system has a degree of robustness to tolerate some change without compromising overall defense. Therefore, an intervention that attenuates such a response may not be beneficial (as suggested by Walsh (2019): “*if it ain't broke, don't fix it*”). The real challenge lies in accurately defining the magnitude of change that would constitute “*broke*” (e.g. as depicted by the margin of “robustness” in Figure 3). Extending this idea, it may be argued that “only when it is “*broken enough*”, *will ‘fixing’ it provide any practical benefit*.” For example, studies examining salivary secretory IgA (sIgA) and URTI have noted that risk is elevated only when salivary sIgA levels decrease by over 60% below an individual's “healthy” baseline values (e.g., Neville et al., 2008; Perkins & Davison, 2022). This emphasizes the need for rigorous methodological “groundwork” to establish baseline levels, but this is rarely done in individual studies. While this “critical” threshold is well established for salivary sIgA, similar evidence is not available for other commonly used markers, making it difficult to interpret the clinical relevance of changes. Taken together, these points illustrate the complexities of the immune system and the limitations of attributing URTI risk according to changes in isolated markers.

## 4 THE ROLE OF NUTRITION

As discussed above, the risk of upper respiratory tract infections (URTI) is influenced by a range of factors (including exposure and immune defense factors, as outlined in Figure 1). The interactions between the various contributing factors (Figure 1) in a “real‐world” setting‐which occur together, in varying degrees at different times‐adds to the complexity of URTI risk. Logically, certain combinations of exercise and sub‐optimal nutrition will interact to reduce host defense and increase URTI risk. However, many previous studies in exercise and nutritional immunology lacked clinically relevant measures (Albers et al., 2005, 2013) that truly reflect host defense. As such, many of the conclusions that historically formed the evidence‐base for consensuses in this field (on the role of nutrition) may be questioned, until we can address them again using such models. This is not to say that such conclusions are incorrect, but that we must be tentative in the interpretations and conclusions based on this evidence. We therefore provide recommendations to achieve this in Section 5 of this brief review.

The role of nutrition in modulating immunity and affecting URTI outcomes has been a focal area of study across exercise and nutritional immunology research. Walsh (2019) noted the traditional approaches in nutritional exercise immunology typically evaluated the potential value of nutrition to reduce illness risk (i.e., prevent illness), versus reducing illness burden (i.e., symptom severity and/or duration), and hence time required to “return to play.” While the second outcome is logical, we also suggest these do not need to be mutually exclusive and in some cases nutritional interventions may work via either, or both pathways (i.e., prevention and mitigation). Importantly, certain mechanisms by which nutritional practices influence infection risk may operate independently of immune system modulation. For instance, some substances exhibit direct antipathogenic effects, such as inhibiting viral attachment, replication, or elimination. Examples include polyphenols (Prabhu et al., 2024; Wang et al., 2020) and zinc lozenges (Hemilä (2017))‐ (*although we note*, *as the effects of zinc lozenges are local*, *and do not require the nutrient to be digested/absorbed*, *we also contend that this is not truly a nutritional intervention in this context*), which can actually exert these effects without directly altering immune markers. These non‐immune pathways provide an alternative mechanism for reducing infection severity and duration, and have key implications for interpreting the outcome of immunity and URTI studies (especially if clinically relevant outcomes are not assessed, and other markers are used as surrogates for this).

### 4.1 Food first but not always food only (Close et al., 2022)

**FIGURE 4:**
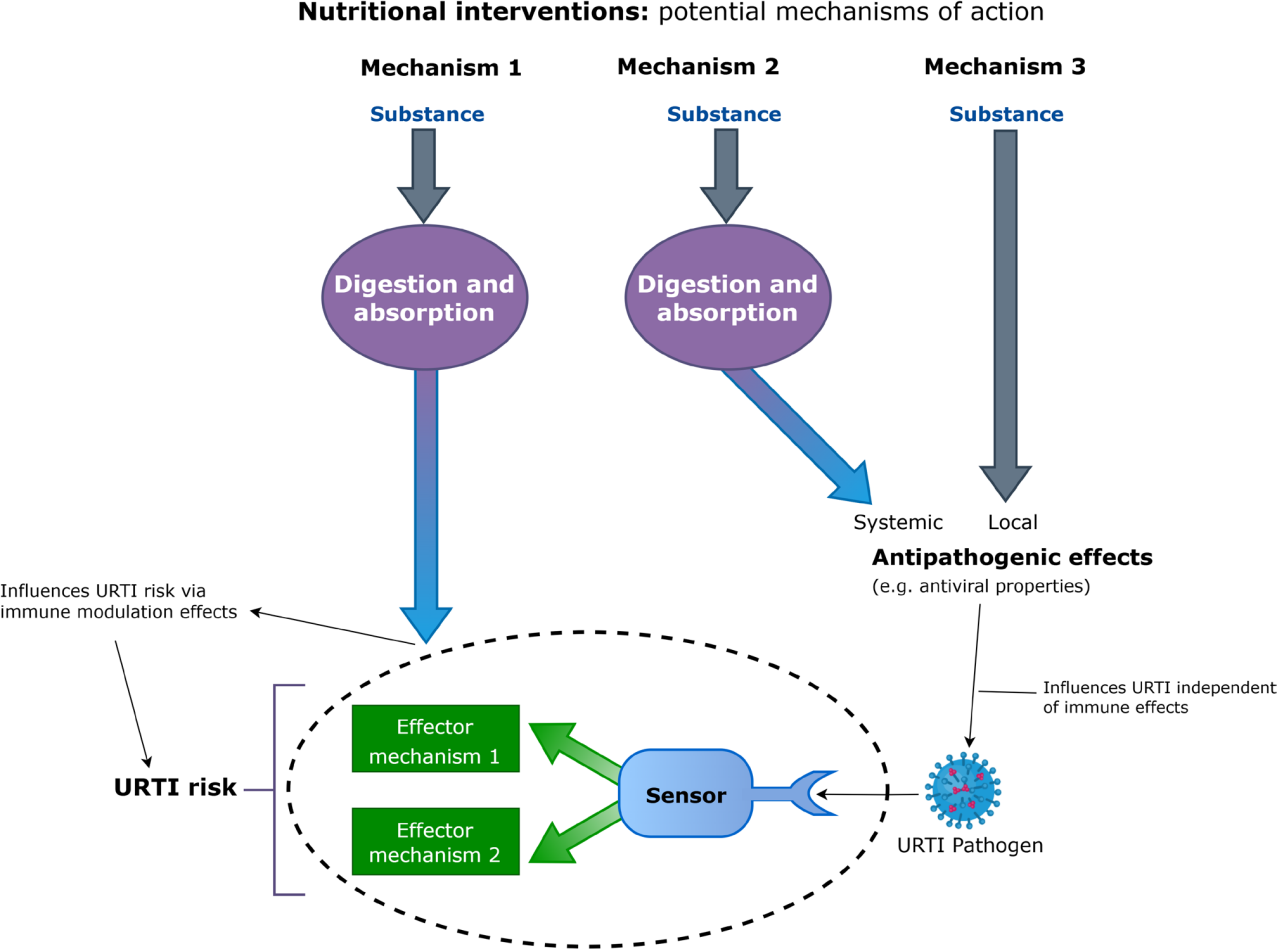
Mechanistic pathways via which nutritional practices or other interventions may influence URTI. Section in oval with dashed outline shows immune mechanisms that may be modulated via this pathway of action (effects may occur either at the level of the sensor, the level of the effector mechanism, or a combination). Mechanisms 1 and 2 require nutrient digestion and absorption and can be considered truly nutritional. Mechanism 3 is local and does not require nutrients to be absorbed (so is not truly a nutritional intervention per se).

The “food first” paradigm (Close et al., 2022) emphasizes the role of diet to prevent nutrient deficiencies. In the context of nutritional immunology, correcting deficiencies would provide benefits to immunity and infection risk compared to the “deficient” state. Often, further supplemental intakes above this provide little‐to‐no further benefit for those without underlying deficiencies. However, failing to assess baseline nutrient status or examine dose–response relationships can lead to misinterpretation of the effects of such supplementation practices (for example, attributing benefits to supplementation when in fact it was caused simply by correcting underlying deficiencies). As such, the most pragmatic approach is to optimize intake via diet, and avoid nutritional deficiency (Close et al., 2022; Davison, Kehaya, Diment, et al., 2016b). Nevertheless, supplementation may be beneficial when dietary preferences or lifestyle factors limit the adequacy of nutrient intake. Furthermore, in some cases additional intake (supplementation) may provide further benefits. Previous consensus statements (e.g., Bermon et al., 2017) and reviews (e.g., Davison, Kehaya, & Jones, 2016a; Walsh, 2018; Williams et al., 2019) provide comprehensive perspectives on nutrition, immunity, and infection risk in athletes, although we must acknowledge that a considerable proportion of the evidence‐base available for such reviews to draw upon suffered from many of the limitations we have noted above. In the following section, we aim to highlight the importance of clinically relevant endpoints, such as URTI presentation, as well as biologically relevant markers, including in vivo immune measures, and issue a call for future research to integrate these approaches, in line with our recommendations, to produce the most robust, reliable and valid evidence in the field of nutritional and exercise immunology. Finally, discrepancies between the observed effects (or lack thereof) of nutritional interventions on immune markers and their impact on URTI risk may be explained by the second and third mechanisms outlined in Figure 4. These mechanisms involve independent antipathogenic effects, such as direct antiviral activity, which are unrelated to immune system modulation (whereas the traditional approaches in this field sought to explain outcomes only through consideration of the first mechanistic pathway in Figure 4); it is important to note that interventions may act via a single pathway, or any combination.

## 5 RECOMMENDATIONS

### 5.1 Conduct studies with clinically relevant measures as primary outcomes

As discussed in Sections 2, 2.1, 3, 3.1, 3.2, 4, there is general agreement within exercise and nutritional immunology (Albers et al., 2013; Bermon et al., 2017) that clinically relevant outcomes (e.g., validated URTI questionnaires, pathogen verified infection, physician identified URTI) and in vivo immune measures are most relevant and valuable. The available evidence in athletes includes clinically relevant measures but often as secondary outcomes (with studies too short or underpowered to detect effects for these), limited pathogen screening/infection verification, limited use of in vivo measures, and use of non‐validated symptom diaries. While ex vivo measures may help advance our understanding of the effects of exercise and nutrition on immunity (and should be included to contribute to mechanistic understanding), there has been an overreliance on such measures (as discussed above). The debates around the relative importance of various factors affecting susceptibility to URTI (i.e., exercise‐induced immune changes vs. other factors) are at least partly due to the widespread imbalance in focus between clinically relevant and ex vivo measures as the primary outcomes in this field. Arguably, research runs the risk of discounting or missing the clinical relevance of interventions if conclusions are based on ex vivo measures alone. For example, take the study of Nieman et al. (2007) where the use of quercetin had no impact on the comprehensive battery of ex vivo measures of immunity but did reduce URTI in the weeks post‐exercise (with [immune‐independent] pathway 2 in Figure 4 being the most likely mechanism in this case)‐ if ex vivo markers had been used alone (without accompanying URTI outcomes), these beneficial effects would have been missed.

Although expensive and logistically difficult, pathogen screening of those reporting URTI symptoms will provide the best evidence, but screening panels should be broad to gain comprehensive coverage of all potential URTI‐causing pathogens and avoid false negatives (e.g., Kustin et al., 2019). Employing multiple swab timepoints (i.e., over several days of perceived symptoms) vs. a single swab is also advised to reduce the likelihood of false negatives and false positives. In addition, quantification of viral load offers further insight into infection status and the effects of interventions, which is not gained by simple positive/negative classifications (e.g., Davison et al., 2025) (note: for most accurate and valid results for viral load, normalization against an appropriate “endogenous” marker is required to account for variations in sample collection/quality, sample processing, and recovery of intended biological material in the swab etc). Perhaps the biggest challenge with free living studies in athletes is the inability to have control over the pathogen exposure rate, timing and “dose,” which will significantly impact URTI occurrence, and symptom severity and duration. This can only be controlled in direct viral‐challenge studies (e.g. Cohen et al., 1991; Prather et al., 2015). However, large sample sizes (i.e. at least *n* > 100), with robust randomization procedures (see Section 5.2) will help ensure similar overall exposure (and pathogen type) risk between intervention and control groups. It is because of the difficulties noted above that high value markers which represent the whole integrated immune response, as a good proxy of host defense per se, are desirable, but they only apply to pathway 1 in Figure 4. For interventions that work via pathways 2 and 3, clinically relevant outcome measures (e.g., validated URTI logs; pathogen screening) are essential.

### 5.2 Ensure an appropriate sample size to achieve sufficient statistical power

Sample size is a key determinant of research design and methodology. The sample size should be large enough to prevent erroneous conclusions, for the effect sizes of interest (Lakens, 2022). Research in this field is often small and potentially underpowered to adequately answer the research question and provide valid conclusions. As an example, to test an intervention's effect on a clinically relevant outcome (e.g., URTI symptoms ratings; episode duration) between two groups (e.g., supplement vs. placebo) using a typical alpha of 5% and 80% power, 128 participants are required (assuming an average of one illness episode per participant) if the expected effect size is medium (*d* = 0.5) and 50 participants for a large effect size (*d* = 0.8) (using an unpaired *t*‐test). As most studies report fewer than 1 URTI episode per participant during monitoring periods, an even larger sample size would be required to account for this. Many typical sample sizes seen in the literature in these fields would only be sufficient to detect very large effect sizes, whereas for many interventions the magnitude of effect may be much smaller than this. Few studies have samples this large, increasing the likelihood of erroneous conclusions (i.e., smaller studies may only be powered to detect very large effect sizes). Our recommendation here is that researchers must determine meaningful effect sizes of interest for their research question and use a‐priori power analysis to demonstrate that the sample size will be adequate to minimize the possibility of erroneous conclusions arising as a consequence of the study being underpowered. Ideally, this will be published prospectively on a publicly available trial register.

### 5.3 Implement appropriate controls and standardization procedures (including nutrition and dietary intake)

As discussed in Section 1, immune defense and URTI risk are influenced by various factors. If these factors are not study variables (either outcomes, or factors being manipulated as part of the study), there is a need to control for their potential confounding impacts. While it is difficult to control all factors associated with infection susceptibility, there are some key ones that must be prioritized, which we overview in the following section.

#### 5.3.1 Pathogen exposure

URTI causing pathogens are transmitted most commonly via unintentional exposure to virus containing secretions, or infected aerosol droplets or particles. The more time spent in shared spaces, and the more people therein, the greater the risk of contracting an URTI. For individuals working in face‐to‐face environments, especially those who work with children, their incidence of URTI is usually increased; likewise, cohabitation, and especially with younger children in the household, and current household URTI, are notable risk factors (Keaney et al., 2021; Keaney et al., 2022; Perkins & Davison, 2022; Petrie et al., 2013). Controlling for exposure variations can be achieved by appropriate randomization and balanced group allocations, accounting for factors like occupation, type of athlete (e.g., individual or team sport; endurance or not), geographical location, young children in the home, etc. Seasonal factors also impact URTI, and should be built into the design to ensure this is not a confounding factor (and is balanced between groups for example).

#### 5.3.2 Participant demographics

There are several characteristics that may influence immune and URTI outcomes, such as age, biological sex, type of athlete, training status, and training load. For crossover studies, where participants serve as their own controls, this is not an issue for some variables, but for other factors that may vary during the study (e.g., menstrual cycle phase, hormonal contraceptive use) controls for these should also be built into the study design. For between‐groups designs, procedures must ensure all relevant factors are balanced between groups.

#### 5.3.3 Lifestyle

##### Nutrition and diet

Nutritional status, acute, and chronic dietary intake affect immunity and infection risk (e.g., Calder & Jackson, 2000). This is even more important if the aim is to determine whether a nutritional intervention is of benefit. Valid conclusions therefore require nutritional status and nutritional intake (acute, and/or chronic depending on study design) to be considered and controlled. Many aspects of dietary intake could influence the outcomes, so these factors should be established and controlled within studies where possible. As an example, studies investigating the effects of an intervention in an acute exercise context would benefit from controlling dietary intake in the 24–48 h before the intervention (and ideally during the subsequent monitoring period). Longer‐term studies should capture periodic records (e.g., several 24‐h food diaries) to determine average nutrient intakes. If captured at the beginning of the study, this can be built into the randomization plan; otherwise, it can be recorded and serve as a covariate during data analysis (although this is not as powerful, e.g., Kang et al., 2008).

##### Other lifestyle factors

There are several other lifestyle factors known to influence immunity and infection risk (e.g., Figure 1), including psychological stress (Cohen et al., 1991; Edwards et al., 2018) and sleep (Prather et al., 2015). While direct control of such factors is generally not feasible, researchers can measure and quantify these factors to incorporate into data analysis (e.g., as covariates). Researchers can also evaluate these measures for potential impact on study outcomes and mitigate as required (e.g., if sleep in the days before each trial visit was recorded, researchers can compare the records before subsequent visit(s) to determine if it is similar enough to proceed or warrants the visit to be rescheduled).

##### Vaccination

Study participants may have different vaccination histories. These differences could influence URTI incidence, severity, or duration. For example, annual influenza vaccinations and COVID‐19 vaccinations would be particularly relevant in this regard. As such, these factors should also be controlled (e.g. incorporated in randomization and group allocation balancing procedures etc. wherever possible).

In summary, to ensure valid and reliable study outcomes, controlling variables that could influence results is essential. This includes randomization, balancing groups, and considering potential confounding factors. Researchers should determine which factors are important to control and employ appropriate procedures for this, to balance influential variables between groups (e.g., age, training load etc), or if not feasible, a less ideal approach is to record them for possible corrections in the statistical analysis. This is most important in small studies (*n* < 100), where imbalances are more likely to emerge in confounding factors without the application of procedures to identify and balance them between groups. In larger studies (i.e., *n* > 100) there is a greater probability of achieving balance across factors by simple randomization.

### 5.4 Conduct longer‐term studies with natural infections or controlled experimental infection challenge

Research studies relying on natural infection face challenges in controlling for sources of variation (e.g., seasonal effects) in exposure to pathogens (see also controls and standardisations section above). Indeed, researchers have purposefully selected the winter period to test the impact of medium‐term (e.g., 12–16 weeks Gleeson et al., 2011; Jones et al., 2014) or short‐term (e.g., 4 weeks, Haywood et al., 2014) nutritional interventions in athletes. Such natural infection studies, however, always carry an element of risk of missing peak URTI incidence or incidence being lower than expected norms in a particular calendar year. In studies aiming to advance understanding of overall risk and URTI incidence between different populations (e.g., endurance vs. other athlete groups) or conditions (e.g., training phases), we recommend longer term (e.g., 12 months) evaluations, similar to that routinely done for studies of viral‐induced exacerbations of airway diseases (Beeh et al., 2009) that account for seasonal variation in infection risk and provide information for other research questions such as the frequency and timing of URTI compared to the general population. We recognize that interventions of such duration would require increased funding and resources but arguably provide more robust and generalizable evidence on the effectiveness of interventions (in particular comparing overall URTI risk between athletes and non‐athletes).

In contrast to naturally occurring infection studies, experimental infection challenge permits a tightly controlled investigation of a single pathogen and facilitates precise timing of interventions and outcomes (symptoms and biological) around infection (Mallia et al., 2011). Most exercise experimental infection challenge studies have been conducted in animals. Relevant studies in this area (e.g., with respiratory infections)‐have shown that short (3 day) periods of prolonged strenuous exercise before or after inoculation lead to worse outcomes, including faster sickness onset and increased mortality (with moderate exercise leading to better outcomes) (Lowder et al., 2005; Murphy et al., 2008). The one published human infection challenge study involved inoculation with rhinovirus (HRV‐16) (Weidner et al., 1998). Within 18 h of first inoculation, a moderately fit student population was randomized to 6 days of supervised moderate‐intensity exercise (cycling, treadmill walking, or jogging) or no exercise. There were no significant effects on symptom ratings or mucous weight (as a surrogate for symptom severity); although this study only examined a very moderate‐intensity exercise intervention, it did not reveal the effects of more strenuous exercise. Nevertheless, human studies have examined other stressors, which appear in Figure 1, such as psychological stress and sleep (Cohen et al., 1991; Prather et al., 2015). Human and mouse experimental infection models effectively complement each other and have broadened our understanding of illness and identified many potential therapeutic approaches (Girkin et al., 2019). Indeed, when considering the above‐mentioned studies together (with all stress types collated) a pattern emerges for worse outcomes in situations of high “stress” occurrence (i.e., either strenuous or exhausting exercise; high psychological stress; lack of sleep) versus lower stress (i.e., moderate exercise or rest; low psychological stress; adequate sleep). Considering the 3Rs of Replacement, Reduction, and Refinement in animal research and with due consideration of the ethical aspects of human studies (Hope & McMillan, 2004), we recommend experimental human infection challenge as an alternative method to long‐term natural infection studies. Such designs can provide clear evidence on the effect of strenuous exercise on URTI and proposed countermeasures.

## 6 SUMMARY: THE CASE FOR THESE RECOMMENDATIONS FOR ROBUST AND RIGOROUS RESEARCH ON NUTRITION, EXERCISE, IMMUNITY, AND URTI RISK

To summarize, previous research in this field on nutritional interventions and URTI risk have often focussed on ex vivo markers. However, the use of isolated ex vivo markers overlooks immune robustness and compensation principles. As such it is unsurprising that interventions affecting these markers do not always align with URTI risk in athletes. Clinically relevant, in vivo markers are more valuable and valid, and seem to have better alignment to URTI risk in such intervention studies. Examples of such discrepancies can be seen for interventions that historically were deemed beneficial according to research with ex vivo measures as the primary outcome. For example, acute carbohydrate supplementation consistently blunts a range of ‘traditional’ ex vivo markers but shows no benefit on clinically relevant outcomes (URTI risk (Nieman et al., 2002)) nor an in vivo measure, challenge‐type method (e.g., Davison, Kehaya, & Jones, 2016a). In contrast, an intervention shown to influenced in vivo immune response (bovine colostrum supplementation: Jones et al., 2019) has shown beneficial effects on URTI risk (Jones et al., 2014; Jones et al., 2016). Together, these findings support the notion that there is strong alignment between in vivo measures and clinically relevant outcomes, which is not necessarily seen between ex vivo measures and clinically relevant outcomes. Of note, this only examines the pathway for mechanism 1 in Figure 4 (with in vivo markers demonstrating “effects on immune function”). For interventions that may exert effects via the alternative pathways of action (e.g., mechanism 2 example: Quercetin, Nieman et al., 2007 study; mechanism 3 example: Zinc Lozenges, Hemilä, 2017) clinically relevant outcomes are essential. If only pathway 1 was examined in such cases, researchers could arrive at an incorrect conclusion regarding their benefits to URTI outcomes. We now need more studies with higher values markers as primary outcomes (e.g., high‐quality URTI reporting methods and/or in vivo markers; pathogen screening) (although complementary ex vivo measures remain important to provide additional mechanistic information) to enhance knowledge and build a more robust evidence‐base that can inform future consensuses on the role of nutritional practices or supplementation (Table 1).

**TABLE 1:** Summary of key recommendations.

Recommendation	Further information	Issues arising if recommendations not followed
1. Studies should have **Clinically relevant** measures as primary outcomes	Focus on clinically relevant outcomes (validated URTI questionnaires; pathogen verified infection; viral load). Screening panels should have broad pathogen coverage to minimize false negatives. Use multiple swab timepoints to minimize false negatives. In vivo immune measures are valuable, but in the context of nutrition studies only assess the immune‐dependent mechanistic pathway. For interventions that may exert effects via other (immune‐independent) pathways, clinically relevant outcomes are essential. Ex vivo measures should be included as secondary outcomes for additional mechanistic insights. Establish robust “healthy baseline” status for participants and meaningful magnitudes of change to account for immune robustness. Avoid relying solely on single markers to account for immune compensation.	A focus on ex vivo immune markers can overlook immune compensation principles and may lead to erroneous conclusions. Focusing solely on ex vivo measures risks missing clinically relevant outcomes influenced by different pathways. Although more valuable than ex vivo markers, in vivo immune measures (without accompanying clinical outcomes: URTI), also run the risk of discounting or missing the clinical relevance of interventions that can influence URTI via different (i.e., immune‐independent) mechanisms.
2. Studies must ensure appropriate **sample size**	Determine meaningful effect sizes for the research question. Use a‐priori power analysis to ensure adequate sample size and minimize erroneous conclusions.	Increasing likelihood of erroneous conclusions (i.e. smaller studies may only be powered to detect very large effect sizes). Increased possibility of “false negatives.”
3. **Controls and standardization** (including Nutrition and dietary intake)	Balance influential/potentially confounding variables between groups through randomization or record them for statistical corrections. Key factors to consider include: Pathogen exposure (occupation, geographical location, seasonal factors, factors related to athlete type and competition, other household members [especially children]). Participant demographics (age, biological sex, type of athlete, training status, training load). Lifestyle factors (nutrition [acute and/or chronic], sleep, psychological stress) Vaccination history. Medication use (and changes) In participants for whom it applies, it is important to control for menstrual cycle phase (i.e., schedule crossover arms in the same phase, or same day on subsequent cycles). Similar principles should also be applied for those using hormonal contraceptives; and individuals using any form or medication that has a cyclical pattern of intake.	Failure to balance or control key variables can compromise study validity. In particular, smaller studies (*n* < 100), without procedures to balance confounding variables between groups have increased risk of imbalances emerging in confounding factors. Imbalances may result in invalid conclusions.
4. Longer‐term studies with natural infections or **controlled experimental infection challenge**	Conduct studies over longer durations (e.g., 12 months) to assess differences (between groups, e.g., athletes vs. non‐athletes) in frequency, timing, and severity of URTI. Experimental infection challenge can provide clear evidence on the effect of strenuous exercise on URTI and to test proposed countermeasures.	Sources of variation (e.g., seasonal effects) in exposure to pathogens are not adequately controlled or balanced between groups (see recommendation 3 above also), leading to erroneous results and conclusions.

*Note*: if studies do not adequately incorporate key design principles, they risk failing to achieve their objectives and may not be able to give valid and accurate determinations of the effects of an intervention (or other factors of focus).

## FUNDING INFORMATION

G.D., C.C., and A.W.J. have previously received research funding from companies that supply nutritional products (G.D. and A.W.J. Neovite Colostrum UK in 2008, 2010, 2013; G.D. and C.C., SunChlorella Corp., Japan, 2013; G.D.‐Volac Whey Nutrition limited, UK, 2022; Nestec Research Centre, Switzerland, 2002) or URTI countermeasure devices (G.D. and C.C.‐Enzymatica AB, Sweden). None of these products are a focus of this review, although some of these studies are cited (Davison et al., 2025; Jones et al., 2014). None of these funders, or any other parties, had any influence on the writing of this manuscript. All authors declare no conflicts of interest.

## ETHICS STATEMENT

This manuscript is a review/recommendations article and does not involve any original data collection using human participants or animals.

## Data Availability

Not applicable‐no original data included in this review.
